# Fidelity, pragmatism and the *“grey line”* in between—exploring the delivery of a pragmatic physical activity randomised controlled trial—a secondary analysis

**DOI:** 10.1186/s12874-024-02242-1

**Published:** 2024-05-21

**Authors:** Abigail J. Hall, Victoria A. Goodwin, David J. Clarke

**Affiliations:** 1 Public Health and Sports Science Department, University of Exeter, Heavitree Road, Exeter, EX1 2LU UK; 2 Academic Unit for Ageing and Stroke Research, Leeds Institute of Health Sciences, University of Leeds, Leeds, UK

**Keywords:** Fidelity, Pragmatism, RCTs, Rehabilitation

## Abstract

**Background:**

Intervention fidelity in health services research has been poor with a reported lack of understanding about what constitutes pragmatic adaptation of interventions and what constitutes failure to maintain intervention fidelity. However, the challenges facing those delivering such interventions have not been thoroughly explored.

The aims of this study were to critically explore the challenges in maintaining fidelity experienced by physiotherapy staff and support workers when delivering a complex intervention for older people living with frailty.

**Methods:**

This study is a secondary analysis of data from a process evaluation of a large randomised controlled trial (RCT). The process evaluation employed qualitative methodologies with mixed methods including a variety of data collection methods, including participant observation, semi-structured interviews and documentary analysis. Thematic analysis was used to make sense of the data.

**Results:**

Many therapy staff felt ongoing confusion about what was acceptable to adapt and what needed to follow the protocol exactly. We found that some therapy staff were able to embrace the challenges of pragmatically adapting interventions while maintaining intervention fidelity, others stuck rigidly to the protocol and failed to adapt interventions where it was necessary.

**Conclusion:**

It was clear that the understanding of fidelity and pragmatism was poor. While pragmatic trials are vital to replicate real world clinical practice, further guidance may need to be developed in order to guide the level of adaptation that is acceptable before fidelity is undermined.

**Supplementary Information:**

The online version contains supplementary material available at 10.1186/s12874-024-02242-1.

## Background

Intervention fidelity in rehabilitation interventions has historically been poor [1], with studies frequently citing challenges such as lack of knowledge, insufficient guidelines and failure of editors to demand assessments of fidelity [2]. Although there are a variety of definitions, the common description is that implementation fidelity is the degree to which interventions are implemented as intended by those who developed them [3]. In physiotherapy research, the need to tailor treatment to individual participants, adapting them to meet the needs of the person often with complex presentations may be considered a challenge [2]. In a recent review of manual therapy interventions for knee osteoarthritis, only one third of included studies were found to have high levels of fidelity [4]. However, it is unclear whether low levels of fidelity in such research relate to the choice of the participant to deviate from the designated treatment, from the therapist delivering the intervention, or more simply, because the fidelity has been poorly reported.

Intervention fidelity is further complicated by the establishment of pragmatic trials. Pragmatic trials are designed to evaluate the effectiveness of interventions in real-life routine practice conditions, whereas explanatory trials aim to test whether an intervention works under optimal situations [5, 6]. Pragmatic trials allow for a level of heterogeneity of the treatment in order to meet the needs of complex and differing needs between participants and settings [7]. Such pragmatic trials are becoming more common place in nursing and health literature [5] and it has been suggested that such studies are the only option when exploring the complexity of population health problems [8]. However, it is recognised that the use of these pragmatic studies may decrease the internal validity of the research [5, 8].

This study seeks to explore the challenges of maintaining fidelity during a pragmatic trial. The Home-based Older People’s Exercise (HOPE) programme is a complex intervention involving a variety of different interacting components [9]. The Home-based Extended Rehabilitation of Older people (HERO) trial was a multi-centre, parallel group, individually randomised-controlled trial with internal pilot and an embedded process evaluation. The HOPE intervention was delivered in two regions of the UK and involved the recruitment of 742 participants. The programme is a 24-week home-based manualised, graded, progressive exercise intervention aimed at improving strength, endurance and balance for frail older people. The programme included a variety of components including behaviour change techniques to aid engagement and adherence. The intervention was delivered by physiotherapy staff and therapy support workers. All therapy staff delivering the intervention received a training session with specific case studies focusing on how to adapt the intervention. These case studies focused on adapting the exercises that were delivered to make them achievable by the participant. A half day training session was provided, therapy staff were then able to attend updates via teleconference held 8–12 weeks.

A mixed-methods process evaluation, informed by the Medical Research Council (MRC) guidance for process evaluation of complex interventions [10] was embedded within the individually randomised HERO trial. This study, a secondary analysis from the process evaluation, explores the challenges of maintaining fidelity of the intervention while undertaking a pragmatic trial, through the lens of the therapy staff delivering it.

### Aims and objectives

The aims of this study were to critically explore the challenges in maintaining fidelity experienced by physiotherapy staff and support workers when delivering a complex intervention for older people living with frailty.

## Methods

The process evaluation employed a qualitative multiple-methods approach which included non-participant observations, semi-structured interviews and documentary analysis. The data for this study were obtained from the interviews with therapy staff and the non-participant observations, which allowed the researcher to observe the delivery of the intervention. Data generation and analysis were underpinned by Normalisation Process Theory (NPT) and drew on the logic model which depicted the intervention (see Additional file 1).

NPT provides a set of constructs which help gain an understanding and explanation regarding the social processes through which practices are operationalized in settings such as healthcare [11]. The theory is constructed of three interlinked processes through which interventions can be explored. It was employed in order to explore the delivery of the intervention by the therapy staff.

The study was reported using Consolidated criteria for reporting qualitative research (COREQ) [12] reporting guidelines (Supplementary file 1). Research ethics committee approval was obtained from HRA Yorkshire & The Humber – Bradford Leeds Research Ethics Committee – reference 17/YH/0097.

### Recruitment

At the therapist intervention delivery training sessions, therapy staff were asked for consent to be involved in process evaluation activities and therefore only participants who had consented were invited to take part. Further informed written consent was obtained prior to all interviews taking place and process consent was obtained from the therapist and participant during the observations.

We purposively sampled therapy staff including a spread across age, gender, level of qualification and years of experience. We stipulated that therapy staff needed to have delivered the intervention to a minimum of three participants to take part in the interview, however, this was a challenge in some sites due to slow recruitment to the trial, as well as high numbers of therapy staff involved in the delivery across time. Sampled participants were contacted via email to determine whether they would be happy to take part in the interview. The participants did not know the researcher prior to the interview, but they may have attended the same training so may have met them prior to the interview.

### Inclusion criteria

All therapy staff who received the HOPE training were eligible for non-participant observations. Therapy staff who completed intervention training and who delivered the 24-week home-based exercise programme to three or more participants were eligible for individual interviews.

### Types of data collected

**Fig. 1:**
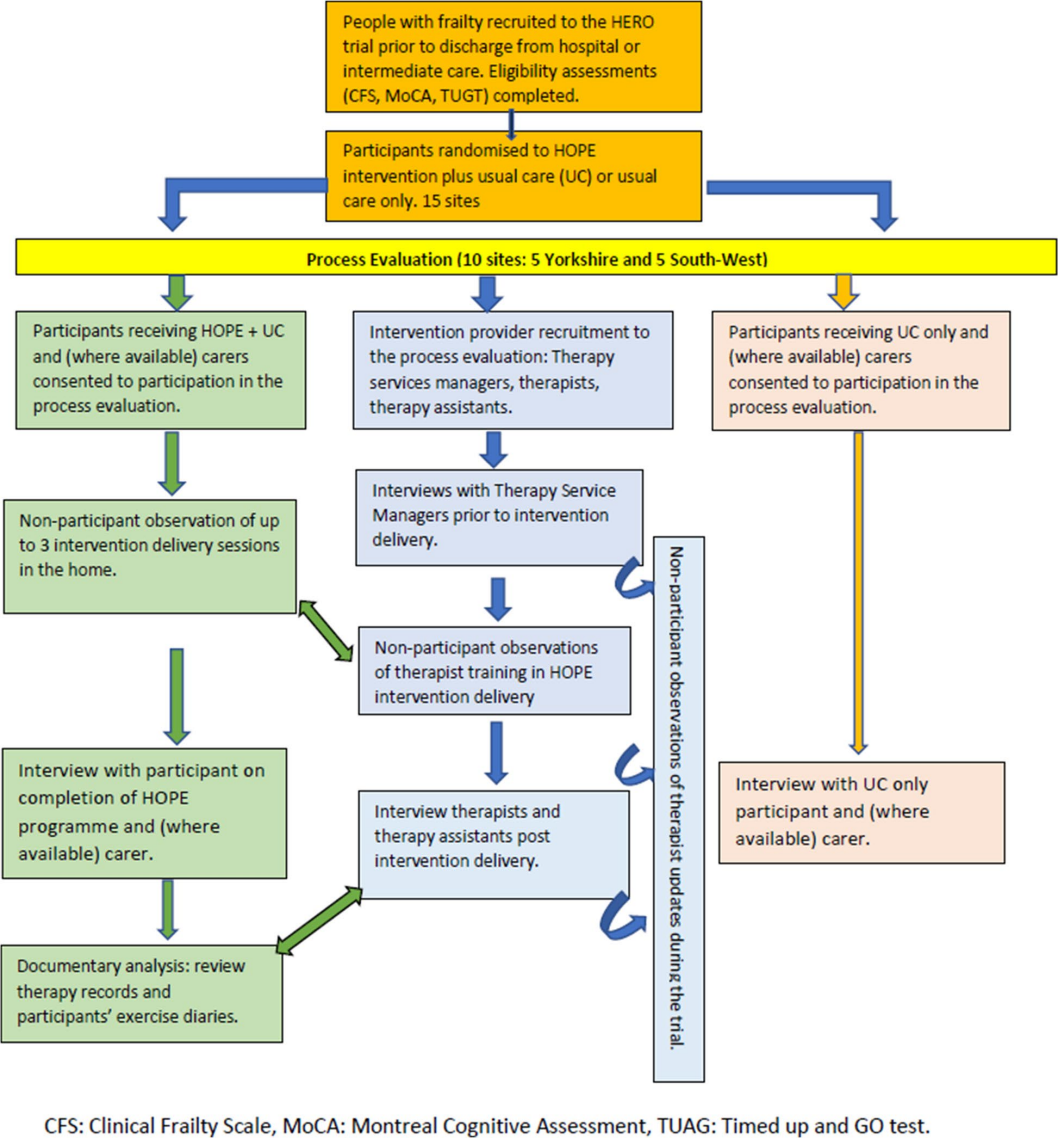
Process evaluation activities (TSM = therapy service manager, HOPE = The Home-based Older People’s Exercise programme, HERO = Home-based Extended Rehabilitation of Older people trial)

Figure 1 depicts the data that were collected for the whole of the process evaluation, with the red boxes demonstrating the sources of data for this study.

### Data collection

Topic guides were developed and used by the researchers when undertaking interviews. The two interviewers (AH and FZ) were both post-doctoral academics with extensive experience of qualitative research, including multiple publications and have both undertaken advanced training in qualitative research. The topic guides were developed using the relevant NPT theory to ensure that the participants’ experiences were explored in relation to the underpinning theory. All data collection tools were created prior to the collection of any data and were piloted with a small number of participants before a final version was agreed and used for the remaining data collection (see supplementary material).

Interviews took place via telephone or face to face and were audio recorded, encrypted and later transcribed. Nobody else was present during the interviews and transcribed by a professional transcriber. All transcriptions were checked and anonymised by the researcher. Field notes were taken where appropriate and the interviews lasted approximately 60 min. At the end of the interview, the participant was asked if they were happy for all their data to be included in the analysis. Sampling continued until sufficient data power was obtained to be able to answer the research aims and objectives.

### Analysis

Thematic analysis [13] was used to make sense of the observational and interview data, adopting both inductive and deductive approaches. The first stage involved the immersion in the raw data generated by all the different types of data collection. The large volumes of data required this to be done using a variety of approaches and in different stages of the data collection and analysis. Summaries were written for all participants including all data relating to that participant as well as data summaries being generated for each type of data. This was done separately by each researcher for each hub and then shared and combined at a later date. This stage of increasing familiarity is a vital step to begin data interpretation [14, 15] and was achieved by listening to recordings, reading transcripts and reviewing field notes. This repeated reading of the field notes and transcripts allowed immersion in the data to increase familiarisation of both breadth and content of the data [16], while beginning to search for meanings or any emerging patterns. NVivo 11 (QSR International) was used to organise data and increase the transparency of data analysis [17]. At all stages scheduled discussions focused on emerging findings from the data analysis ensured researchers were familiar with the data from each hub. This also enabled identification of similarities and differences within and between the two hubs. This process was ongoing throughout the stages of analysis to ensure rigour and consistency of analysis.

An initial stage of coding was undertaken following completion of data collection for each type of data. This coding was undertaken by each researcher independently and then compared across sites. The two authors discussed and compared coding strategies and resolved any disagreements. Following this process, preliminary codes for each set of participant data were generated. A third researcher was involved at this stage in order to develop the codes and consider their application to different data sets.

In order to generate themes, further analysis was carried out individually by researcher in each hub followed by team discussion and consensus building around emerging themes. These themes were further clarified in the context of the logic model underpinning the intervention. Evolving themes were compared between the two hubs/regions to determine any differences or similarities. Data for each of these themes was collated from each type of data and by triangulating this data, it was possible to explore whether different types of data supported each other. For example, reviewing the therapy record for a particular participant’s therapy session was compared to the observational data to determine whether there were any discrepancies in the data.

The secondary analysis involved re-reading of all interviews conducted with therapy staff and observations of home-visits where the HOPE intervention was delivered. An approach based on directed content analysis [18] was utilised whereby findings relating to fidelity to protocol from the process evaluation were used to identify for interview comments on or field-note instances relating to fidelity and/or pragmatic adaptation. These instances were then grouped according to the extent to which they represented delivery as intended according to the HOPE protocol, evidence of adaptation of the intervention to meet participants’ needs or uncertainty in relation to the extent which the intervention could be adapted. Therapy staffs’ comments on factors influencing their actions in terms of adhering to the protocol or making pragmatic adaptations were reviewed and commonalities and differences in approach identified. The secondary analysis was conducted by a single researcher (AH), findings were then reviewed with an experienced qualitative researcher (DJC). The agreed findings were then reviewed by and discussed with researchers VG and AF, members of the HERO trial team.

## Results

Data collection was undertaken in two recruitment hubs (five sites in each), in Yorkshire and the South West. Twenty three therapy staff (Table 1) were interviewed as part of the process evaluation and a total of 61 treatment sessions were observed across all trial sites. All the therapy staff who were approached agreed to take part in the interview and none withdrew.

**Table 1:** - characteristics of therapy staff (NHS Banding – 2, 3 and 4 are unregistered staff, with increasing band representing increased levels of seniority)

Characteristic	**Number (*n* = 23)**
Age	
16–24	2
25–34	4
35–44	8
45–54	5
55–64	3
Not reported	1
Sex	
Female	17
Male	5
Not reported	1
NHS Banding	
2	1
3	0
4	3
5	0
6	14
7	5
Length of experience (years)	
0–5	7
6—10	5
11–15	5
16—20	5
21—25	0
26- 30	1

Secondary analysis suggested that therapy staff could be categorised into one of three different groups when considering their approach and levels of pragmatism adopted. These were those who embraced pragmatism and fidelity and had a clear understanding of how to adapt the intervention, those who delivered the intervention exactly as per the protocol without deviation and a final group who reported ongoing confusion about what could and could not be adapted.

### Embraced pragmatism and fidelity

Less than one third of therapy staff who we interviewed reported having a clear understanding about how to pragmatically adapt the intervention while also maintaining fidelity. These therapy staff were all experienced Band 6 or 7 therapy staff with at least ten years post-graduate experience and all worked in community settings on a day to day basis.

The training that therapy staff received encouraged adapting exercises if the participant was unable to undertake them. There were examples of the therapist altering the exercise to make it achievable where certain elements were not possible.

*So I've had to adapt some of the exercises. I've had a lady who had a problem, has had long-term shoulder problems, so there is a wall press exercise… she couldn't lift her arms up quite enough to get the position. So we sort of slightly adapted her shoulder position to allow her to still do it (participant 109, physiotherapist)*

Instead of adapting the exercise, more commonly, exercises were left out when a participant was unable to manage it.

*But I must admit…… a couple of them couldn’t do one or two of the exercises, so we’ve just kind of left those ones out and carried on with the rest of them, but obviously I’ve documented that. And it seems to be some of the heel raisers, you know, and toe raisers that they have trouble with. (participant 38 , physiotherapist)*

Observational data supports this when, on occasion, exercises were missed out where the participant was unable to do them. (Observation_07885_338_visit 5).

*“Trunk twists are next, but [therapist] reports they aren’t doing this as it causes her back to hurt. [therapist] flicks past this in the manual. “*

Other adaptations included suggesting taking extended time off from the intervention where the participant was not available or had other prior engagements. This was used to encourage the participant to continue in the trial rather than withdraw.

*Yeah, just, you know, yeah like you said, flexibility. I had one lady…she was worried because she went off on holiday and I was like well, don’t worry about the exercises on holiday, take that week off…. and I’ll call you again in a couple of weeks. (participant 91, support worker)*

Reducing the number of times that the exercises were to be completed each day seemed a more common approach that several therapy staff reported in order to prevent complications of the suggested dose of the intervention.

*…….. trying to do three a day would probably just flare it up and just wouldn’t do anything, so you’ve got to sort of keep it at a level (participant 111, physiotherapist)*

While these therapy staff felt confident to make such adaptations without any apparent support, other therapy staff reported the need to seek support and approval to make similar changes. This support was reported to be gained from therapy service managers, other colleagues involved in the HERO trial, attending therapist update meetings (regular teleconference meetings that therapy staff could attend to ask questions about delivery and receive updates about the trial) or speaking directly to the trial manager.

During the course of their involvement in the trial, these therapy staff reported increased confidence to adapt the intervention in the future.

*…. to start with, very much I was trying to do it by the book, like you must do it, and then all this information, and coming out thinking oh they’ve just like given them all that information and I can imagine they’re feeling a bit like what am I doing, so I’ve tried to spread it out a bit more and every time just reiterating the most important parts (participant 003, physiotherapist)*

However, this was reported to take time to develop confidence to be able to adapt the intervention. Often this confidence developed with more participants that they delivered HOPE to.

*Not really, I mean I think you could do what you wanted, well you could and you couldn’t, I thought it was quite regimented at first and as I went along I thought, well you probably could do that, but it’s quite hard to get all those exercises done whilst you’re going and make a cup, there’s a lot to do, whereas if you have three or four then, and I might say to people, you know, “You can pick and choose some of your exercises,” whereas they wanted them in that order, that was the other thing, there was a specific thing to do them in that order, and I don’t know why (participant 19, physiotherapist)*

Most adaptations were small, relating to altering the dose or the way the exercise was delivered, however, there were therapy staff who made more substantial alterations to the intervention. The nineteen telephone contacts scheduled as part of the intervention were viewed variably by participants and therapy staff during the intervention. Two therapy staff reported changing the schedule of the intervention to reduce the reliance on this method of contact.

*I didn’t ring him every week because he didn’t need me to….. I couldn’t catch him sometimes because he was here, there and everywhere. (participant 003, physiotherapist)*

Or they were not undertaken simply because the participant was reluctant to use the telephone so face to face contacts were initiated instead.

*had one lady who didn’t like using the telephone so I wasn’t able to do telephone follow-ups, so I did, I did a three-weekly face-to-face visit instead (participant 61, physiotherapist)*

The Timed Up and Go test was designed to be a fundamental part of the intervention – guiding the therapist as to when the exercise level could be increased or decreased. However, several therapy staff decided that this was not an effective method of progressing their participant.

*the lady with the cellulitis legs, she’s never ever going to be any better at doing a timed get up and go test purely because of her legs, but she’s strong in her arms and when she’s sat down she’s great doing her exercises but she’ll never progress from Level 1 exercises to Level 2 because she’s, I think it takes her 3 minutes to do a timed get up and go test, so she’ll never meet the criteria to go onto the next one but I think she would manage with the next exercises. (participant 33, physiotherapist)*

#### Delivered exactly as protocol

Many of the therapy staff who delivered the intervention reported delivering it exactly as the protocol dictated, with no alteration of the components of the intervention. These therapy staff were band 2, 4 or 6 with the majority having less than 5 years’ experience, although one of these therapy staff did have considerably more than this, they were the exception.

*Not so much. Sometimes we had to anyway because of staffing levels, we had to be a bit flexible, but I felt that there wasn’t an awful lot of room to manoeuvre, just the way that it was all set up (participant 21, support worker).*

The methods of progressing participants were reported to prevent them changing the level for the intervention in some cases. For example, therapy staff in this group felt that the participant could progress a level, but was restricted by the Timed Up and Go and therefore did not feel able to progress them.

*Because they were doing really, really well within level one and they could move like, they could definitely do the level two exercises, but it was the timed up and go that was the restricting, to say, ‘this is your level’ (participant 102, physiotherapist)*

Reasons for delivering exactly as per protocol related to understanding of trial protocols whereby the intervention had to be delivered to all participants in an equal fashion, despite the opportunity to deliver it pragmatically.

*I did feel I have to follow procedures because I felt, and my understanding of the trials says you kind of have to you know, put certain rules, like structures, and if you’re going to go away on a tangent almost then you know what you deliver to one person will be different to another person so where is the objectivity of this and the whole trial? (participant 63, physiotherapist)*

Other therapy staff were very clear that their role was to deliver the intervention exactly as per the protocol and had a very clear remit. These therapy staff worked primarily in acute settings and were not routinely working in community-based settings.

*‘that wasn’t what we were there to provide, like they made it very clear that, we need to make it clear ourselves that we’re not a community therapy team, that’s not our role, we’re here to be part of the research trial and do, this is what we teach, this is our remit as such. (participant 102, physiotherapist)*

While some therapy staff did not question the elements of the intervention and delivered exactly as planned, other therapy staff questioned various components and reported concerns that the intervention was not entirely suitable for the participant, but they continued to deliver it without alteration. Where the therapist followed the protocol without alteration, there was a feeling of dissatisfaction and suggestion that their skills as a physiotherapist were not required to deliver the intervention.

*I just didn't feel like a physio, that was the only thing with him, I didn't feel like I'd been me. I felt like I could have been anyone, and, theoretically you could have been a non-qualified member of staff doing that. And I just mean that when you're reading from a book, it feels very stunted and very much how you, when you first start as a physiotherapist you have no clinical experience (participant 109, physiotherapist)*

Whereas several of the more experienced therapy staff questioned the value of certain aspects of the intervention, such as the telephone contacts, and omitted some, other therapy staff continued to deliver according to the schedule despite feeling there was no benefit to the patient with certain elements.

*… you can tell they’re engaged on the phone and …., it does feel very repetitive for the ones that you just think, you’re logging the same thing, every week you know (participant 144, support worker)*

While some therapy staff reduced the dose of the intervention delivered, others felt that the dose was too high, but still encouraged their participant to stick to the required dose in order to maintain fidelity.

*I think that is too big a commitment.………even the person who was very motivated and did a lot of the exercises, he struggled, which he said, uh, it’s a lot, you know, three times a day is difficult to fit in, and it’s a lot (participant 23, physiotherapist)*

Two of the physiotherapy staff interviewed reported that they were uncomfortable delivering an exercise intervention without undertaking a full physiotherapy assessment. Despite appreciating that this was the nature of the intervention and the participant had been screened for suitability for the trial, there was noted discomfort in the approach they were having to take as part of the trial.

Observational data supports the delivery by some therapy staff in which the protocol was followed exactly. There were examples of non-participant observations where participants were noted to be struggling with certain aspects of the exercise programme, however, the therapist failed to take the opportunity to adapt the intervention, suggesting they just “keep trying”.

### Ongoing confusion

The final group of therapy staff reported ongoing confusion with what could be pragmatically adapted and what was needed to be delivered to maintain fidelity. This included all bands of staff and levels of experience, although there was some suggestion that those with greater experience of working in the community seemed more confident to adapt the intervention to suit the environment, context and the participant. These therapy staff often sought advice from the trial manager, other colleagues delivering the HOPE intervention or, in some cases, the researchers undertaking the non-participant observations. Despite this support, their confusion never seemed to be resolved. There was also some suggestion that the level of adaptations allowed varied during progression of the trial. Initially therapists reported that the intervention appeared quite rigid, but the therapist updates allowed therapists to discuss and share practice, which highlighted greater flexibility to adapt the intervention. It was also evident that as therapist experience of delivering the intervention grew, so did their confidence to adapt the intervention.

Many of the therapy staff reported a poor memory of the training that was delivered as part of the HERO trial – often as the training had far preceded the recruitment of their participants. The extent to which adaptations were discussed in the training was reportedly variable in different sites, but what was a common theme was the lack of clear understanding of adaptable components and which components of the intervention must not be adapted.

*Well it might do, and it might be how the training was, do you know what I mean, because the training was quite this is how you do it, so I didn’t really feel that they said in the training that you had a lot of…...I thought we had to be quite rigid in how we did it, this was a research … because you’re going to be looking at it from a research point of view, that why would you vary it, because how would that show, do you see what I mean? (participant 19, physiotherapist)*

Where therapy staff did adapt the intervention, they were often unsure whether they were meant to be doing so. This lack of confidence was further demonstrated when during non-participant observations, the therapist often asked the researcher if they were allowed to change things. This concern was palpable that therapy staff weren’t sure how much they could adapt without affecting the outcome of the trial.

*we eventually got him doing sort of like one, he started again once a day, then twice a day, then three, because he couldn’t sort of psychologically manage doing three times a day one bit so, but then you realise you’re not sort of fulfilling the spec as it were of the actual trial. And then you’re thinking well how is that going to be analysed in terms of the outcome measures? So that was a bit difficult. (participant 111, physiotherapist)*

While some therapy staff saw their role to deliver the intervention as planned and directed patients to other community services such as their GP when they needed input that fell outside the remit of the trial, others felt that it was their duty to deliver what the participant needed. A common theme amongst the therapy staff was reportedly the need for participants to practice climbing stairs and general mobility practice. While some therapy staff reported they had declined to do such activities as they weren’t part of HOPE, others reported they felt they should undertake such treatments. There was particular confusion when the goals set by the participant included such activities.

Where there were challenges in the participant managing to undertake the exercises, some therapy staff suggested trying additional adaptations to make it easier for the participant to engage, although they felt this was morally the right thing to do for the patient, it led to further confusion about whether they should have undertaken such adaptations.

*And we worried that potentially she wouldn't be doing the exercises quite as much, and the fact that I had to write on the diary the dates and almost make her a little fake sheet at the front and go through it again how to do it, and highlight, using a highlighter, which, again, is not part of the protocol. But I actually, rather than just star the exercises, I highlighted the exercises that she had to do and had to make quite a few adaptions to it. The trial doesn't necessarily allow for those kind of ada... or it doesn't suggest those kind of adaptions, partly because it's a trial. If it was rolled out, I can't see why adding colour and adding stars and, you know, writing things out for people, is a negative. But I think, based on the trial, you'd probably skew the data if you did that too much, if one person chooses to put lots more interventions in. (participant 109, physiotherapist)*

These narratives demonstrate that the level of adaptation allowed remained unclear for some therapy staff throughout the trial and therapy staff continued to deliver the intervention but were not clear whether the adaptations they were making would affect fidelity. Interestingly, there was no evidence from those receiving the intervention that therapy staff were unsure about how to deliver the intervention. However, observational data supports this ongoing confusion that some therapy staff felt. Both researchers undertaking non-participant observations of the intervention reported therapy staff asking them to clarify aspects of the intervention and delivery during these non-participant observations.

## Discussion

The aim of this study was to critically explore the challenges that physiotherapy staff and support workers experienced balancing delivering a pragmatic intervention while maintaining fidelity of a complex physiotherapy intervention designed for frail older people. This was a secondary analysis of data from the process evaluation, undertaken due to a recurring theme that became evident during non-participant observations and again in analysis of the interview data.

We found that less than a third of the therapy staff we sampled were confident to appropriately adapt the intervention while maintaining a clear understanding about how to ensure fidelity of the intervention. The remaining therapy staff either adapted the intervention according to the needs of the participant (although there is suggestion that some adaptations were beyond what was expected), or delivered the intervention without adaptation as they perceived this was necessary to ensure that fidelity was maintained. The extent to which the adaptation demonstrates a pragmatic approach which was encouraged by the trial or actually reduces the fidelity of the intervention must be considered. However, more importantly, it represents the challenge that some therapy staff felt being told that the intervention should be delivered pragmatically – how far they were able to go to adapt the intervention while maintaining fidelity. Observational data supports the interview data and demonstrates that different therapy staff adapted the intervention to different amounts – ranging from changing the number of repetitions, to omitting exercises, to changing the delivery schedule of the planned contacts.

During the training that all those delivering the intervention received, case studies were used to help therapy staff consider adaptations to the intervention, but these case studies involved adapting the actual exercises rather than changing the dose or the schedule of delivery. While emphasis was placed on the pragmatic nature of the intervention during training and update sessions, there was little discussion about what could or, more specifically, could not be adapted.

Pragmatic randomized clinical trials are becoming increasingly common in order to break down the barriers of translation of interventions into practice [19]. RCTs involve the creation of highly controlled conditions to evaluate an intervention’s efficacy, whereas pragmatic RCTs aim to evaluate an intervention’s effectiveness in “real world” situations where such conditions cannot be completely controlled [19]. The challenge that our participants demonstrated was trying to reason which elements of the intervention could be adapted accordingly. The term “flexible fidelity” has been suggested to describe the need to deliver core elements of an intervention while allowing for the purposeful adaptation of non-essential intervention elements [20, 21]. The challenge that we noted was the confusion surrounding what elements were deemed essential and which were not. Initially, participant retention in the trial was poor with many participants dropping out. There was suggestion that this led to the trail team encouraging therapy staff to adapt the intervention more to ensure that participants continued the intervention. This led to confusion amongst the staff delivering the intervention.

Few studies have attempted to review the literature on pragmatic RCTs [22] and those that have are suggested to rely on weak inclusion criteria such as the use of the term “pragmatic” in the title or abstract [23]. While there is a plethora of research utilising a pragmatic RCT methodology, to our knowledge, this is the first study which explores the challenges that those delivering the interventions face. To ensure that interventions are delivered as planned, it is important to consider the experiences of those delivering such interventions. Our data highlights the challenges that clinicians face when delivering a complex intervention with multiple components to a population often with multi co-morbidities as part of a pragmatic RCT. We propose that essential components of a pragmatic trial are made explicit to those delivering the intervention. These components should not be adapted to maintain fidelity. However, the non-essential components of interventions, which do not compromise fidelity, can be adapted to meet individual and local context. These concepts of fidelity and adaptability are associated with the scaling up interventions for spread and adoption [24]. There is also suggestion that the confidence of clinicians participating in a research trial – and their understanding of research processes – could be improved and this may itself increase their ability to pragmatically deliver research interventions.

## Conclusion

Our study suggests that we cannot rely on the assumption that complex interventions are delivered as planned. They are especially difficult to deliver when the population often have co-morbidities and contextual factors that affect delivery. It is vital to assess what has been delivered to ensure that this is translatable to everyday clinical practice. While pragmatic trials are vital to replicate real world clinical practice, further guidance may need to be developed to guide the level of adaptation that is acceptable before fidelity is undermined.

### Supplementary Information

Supplementary file 1.

Supplementary file 2.

## Data Availability

The datasets used and/or analysed during the current study are available from the corresponding author on reasonable request.

## Abbreviations

COREQ: Consolidated criteria for reporting qualitative research
HERO: Home-based Extended Rehabilitation for Older people
HOPE: Home-based Older People’s Exercise
GP: General Practitioner
NPT: Normalisation Process Theory
PE: Process evaluation
RCT: Randomised controlled trial
TSM: Therapy service manager
