# Supplementary material for: Fidelity, pragmatism and the “grey line” in between—exploring the delivery of a pragmatic physical activity randomised controlled trial—a secondary analysis
Source: BMC Med Res Methodol. 2024 May 21;24:118. doi: 10.1186/s12874-024-02242-1 (PMC11106995; doi:10.1186/s12874-024-02242-1)
Supplement: Supplementary file 1 [file 12874_2024_2242_MOESM1_ESM.doc]

**HERO Process Evaluation**

**Intervention Participants**

**Interview Topic Guide**

Duration: 60 -75 minutes

**Introduction:**

*Thank you for taking part in the HOPE programme. As I indicated (in my letter/on the phone) at the same time as agreeing to take part in the HOPE programme you kindly agreed to participate in an interview with us. The purpose of our conversation today is to learn a little bit about yourself, to understand your experiences of the HOPE programme and how it was delivered, and to find out if you have any suggestions of how the programme could be improved in the future.*

*Have you had a chance to read the information sheet? Are there any questions you wish to ask about the interview or this part of the study before we start?*

*With your permission I will be audio- recording the conversation, but I just wanted to reassure you that everything you say will be anonymised. That means we will not use your name or any other details about you in the reports or publications we write.*

*If you wish to have a break during the conversation, please say and we can take a break at any time.*

*Just to give you an overview of what I will be talking to you about; we will start by talking a bit about yourself and how you got involved in the programme; then we’ll try and take you back to the beginning of the programme and the therapist visits, what it was like and how you felt about it. After that we’ll discuss how you got on with it as the weeks passed, and last of all we’ll discuss what you have got out of it for yourself, and what improvements we could make to the programme if you have any suggestions. Is that ok?*

*Shall we start? OR Are you ready to start?*

| ***So, before we discuss your involvement in the HOPE study I would like to ask you a couple of general questions about yourself:*** | | | |
| --- | --- | --- | --- |
| Q1: How would you ***describe yourself*** as a person?  Prompt: Has that changed as you have got older?  Prompt: Have you or someone else ever seen yourself as frail? | Q2: Do you think it ***is important to be doing exercise/s*** as people get older?  Prompt: How important do you feel is it for you personally to remain active?  Prompt: Is that something you have always believed?  Prompt: Do you think people can learn new things at any age? | | Q3: Did the therapist who introduced you to the HOPE programme talk to you about the ***importance of exercise*** for older people?  Prompt: Do you remember what they said? |
| ***Turning now to the HOPE study and your experiences of undertaking the exercise programme:*** | | | |
| Q4: Can you tell me why you ***chose to take part***?  Prompt: Did anyone else encourage you to take part, and if so how, and for what reason? | Q5: What do you think the HOPE programme exercises were designed to achieve? | | Q6: What were you (or family members) ***hoping to achieve*** by participating in the study?  Q8: Was the ***therapist’s explanation*** of the programme and the exercises clear?  Prompt: Could they have done anything differently or better? |
| Q7: When you first started the programme what did you think was ***expected*** from you?  Prompt: How did you feel about the programme or the exercises in the beginning? | | |  |
| ***I want to talk to you now about some of the practical aspects of the programme of exercises. If we can talk first about the discussion you had with the therapists about the things that were important for you to achieve- we called these your goals in the study:*** | | | |
| Q9: Can you tell me a little bit about how you and the therapist decided on you***r goals***.  Prompt: Can you recall why you chose those goals?  Prompt: Was the therapist in setting them?  Prompt: Were the goals what you really wanted to achieve or was there some compromise?  Prompt: Did you feel you could achieve the goals easily, or that it would take a bit of effort?  Prompt: Did you ever ‘cheat’ on filling in your diary? [Alternative: Did you always complete the diary accurately?] | Q10: Did you ***change any of your goals*** during the programme?  Prompt: If so, how did you go about changing the goals with the therapist? | | Q13: Did you ***identify any rewards or incentives*** with the therapist?  Prompt: Were they useful in motivating you along the way? |
|  | Q11: How useful did you find ***the discussion of your goals*** with your therapist when they visited you at home?  Prompt: And when they telephoned you?  Prompt: Was it the same person? Was there a difference? | | Q14: Did you ***ever feel anxious*** that you couldn’t achieve your goals?  Prompt: or anxious about other aspects of the programme?  Prompt: What did your therapist say or do to help or support you |
|  | Q12: Were you able to achieve the goals you agreed? If so, how did that feel? | |  |
| ***If we can talk about doing the exercises themselves now:*** | | | |
| Q15: What did you think of the ***exercise manual?***  Prompt: Text, language, pictures…  Prompt: Did you document your goals  Prompt: Were there any specific exercises that you liked/didn’t like?  Q16: How did you find ***learning the exercises?***  Prompt: Did you have any difficulties remembering the exercises? What did you do to help?  Prompt: Did the fridge magnet, pens and bag help to remind you? | | | Q17: How did you find the ***exercise diary?***  Prompt: Did the therapist give you instruction on how to complete the diary?  Prompt: Do you complete the diary every day?  Prompt: Did the diary help you to see your progress? |
| Q18: When you started the exercise programme, do you remember if your therapist talked to you about what ***to expect in terms of aches or pains***?  Prompt: How did you feel about that at the time? | Q19: As ***you started doing the exercises*** how did you feel?  Prompt: Did you experience any difficulties or ever feel like giving up the exercises?*  Prompt: Were you able to discuss these issues with the therapist?  Prompt: Did your therapist support you with those?* Prompt: Did anyone else support? | | Q20: In your first or second session did the therapist discuss with you how to ***exercise safely***?  Prompt: Do you recall what they said or what they showed you?  Prompt: Did they or you make any changes in your home to improve your safety?  Prompt: How did you feel about making those changes? |
| **Now we are interested to hear about how the exercises fitted into your daily life:** | | | |
| Q21: Did ***your therapist help you to plan*** the exercises into your daily routine?  Prompt: How did this work out? Did you have to make adjustments?  Prompt: Did you feel that the therapist listened to you and was then able to adapt the exercises to fit in with your routine  Prompt: How did the therapist help you to keep up your exercises as much as possible | Q22: Did you discuss ***how and where*** you would do the exercises with other people/ family members? | | Q23: How did your ***therapist progress or change*** the exercises over the months you worked with him/ her? |
|  | Q24: Did you use the ‘staying on track’ exercises? Did your therapist explain to you when and how to use these exercises? | | Q25: Did ***you ever make any changes*** to the plan yourself?  Prompt: If so, what did you change? How did it work out? |
| ***I want to ask you now about the different way the therapist worked with you whilst you were completing the exercises:*** | | | |
| Q26: How did you find ***the five home visits*** with the therapist?  Prompt: Were there any issues with organising the visits?  Prompt: In your face to face contacts with the therapists did you discuss your progress?  Prompt: Did you feel that you could be honest with the therapist if you were struggling?  Prompt: If you were struggling, what advice or solutions did the therapist offer you? Did you come up with a solution yourself? | | | Q27: What about ***the telephone call*** from the therapists- did you find those helpful?  Prompt: Were there any issues with organising the phone calls?  Prompt: Was the therapist supportive?  Prompt: Was it the same person as in the home visits? Did that matter?  Prompt: Did the phone call keep you motivated? |
| ***We know that support from other people around you can be important in continuing to do exercises like those in the HOPE programme. I have just a few more questions about that now:*** | | | |
| Q28: Were ***any members of your family or your friends involved*** in helping you with the programme in any way?  Prompt: Did you ask them or anyone else for help? If so, were they supportive | Q29: Were any members of ***your family involved deciding on goals*** or thinking about incentives to do the exercises?  Prompt: Did any members of your family take part in the exercises with you?  Prompt: Do you know anyone who does similar exercises?  Prompt: Did you discuss your participation in the research, or the exercise programme with other people, friends or neighbours | | |
| ***We have now come to the last couple of questions; these focus on your thoughts about participating in the programme and what we might do to improve on the programme for other people:*** | | | |
| Q30: Have you ***enjoyed taking part*** in the programme?  Prompt: What do/did you particularly like?  Prompt: What do/did you get out of it for yourself?  Prompt: If they supported you, do you think your family members thought it was helpful for you to take part in the programme | Q31: Do you think you will ***continue with the exercises*** now that the programme has finished?  Prompt: What other goals do you have for the future | Q32: Do you feel that the ***exercises make a difference*** to how you feel or what you are able to do?  Prompt: Was it worth the effort?  Prompt: Have the exercises improved your confidence | |
| Q33: Did or do the ***exercises fit in*** with the rest of your daily life?  Prompt: If it does not fit can you explain some of the reasons?  Prompt: What would help you to exercise more regularly?  Prompt: Did or does the programme help with any other aspirations or wishes you have? | Q34: How do you feel about the ***length*** of the programme?  Prompt: There were five home visits and nineteen phone calls  Prompt: Have your thoughts on the length of the programme changed as you have gone through it? | Q35: Overall***, do you think the programme is well thought out***?  Prompt: Are there things you think we might improve for other people like yourself in the future?  ***And the last question is:***  ***Q36: Do you have any further comments or is there anything we have forgotten to talk about?*** | |

*Thank you very much for your time. I really enjoyed talking to you.*
